# Aberrant IL-17 Levels in Rodent Models of Autism Spectrum Disorder: A Systematic Review

**DOI:** 10.3389/fimmu.2022.874064

**Published:** 2022-06-10

**Authors:** Alexandra Jade Thawley, Luciana Peixoto Veneziani, Francisco Diego Rabelo-da-Ponte, Ingo Riederer, Daniella Areas Mendes-da-Cruz, Victorio Bambini-Junior

**Affiliations:** ^1^ School of Pharmacy and Biomedical Sciences, University of Central Lancashire, Preston, United Kingdom; ^2^ Laboratory on Thymus Research, Oswaldo Cruz Foundation, Oswaldo Cruz Institute, Rio de Janeiro, Brazil; ^3^ National Institute of Science and Technology on Neuroimmunomodulation (INCT-NIM), Oswaldo Cruz Institute, Oswaldo Cruz Foundation, Rio de Janeiro, Brazil; ^4^ Rio de Janeiro Research Network on Neuroinflammation (RENEURIN), Oswaldo Cruz Institute, Oswaldo Cruz Foundation, Rio de Janeiro, Brazil; ^5^ Laboratory of Molecular Psychiatry, Centro de Pesquisa Experimental (CPE) and Centro de Pesquisa Clínica (CPC), Hospital de Clínicas de Porto Alegre (HCPA), Porto Alegre (RS), Brazil; ^6^ Division of Biomedical and Life Sciences, Faculty of Health and Medicine, Lancaster University, Lancaster, United Kingdom

**Keywords:** IL-17, autism spectrum disorder, animal model, systematic review, inflammation

## Abstract

Autism spectrum disorder (ASD) is a heterogeneous neurodevelopmental disorder characterised by stereotyped behaviours, specific interests, and impaired communication skills. Elevated levels of pro-inflammatory cytokines, such as interleukin-17A (IL-17A or IL-17), have been implicated as part of immune alterations that may contribute to this outcome. In this context, rodent models have helped elucidate the role of T-cell activation and IL-17 secretion in the pathogenesis of ASD. Regarding the preclinical findings, the data available is contradictory in offspring but not in the pregnant dams, pointing to IL-17 as one of the main drivers of altered behaviour in some models ASD, whilst there are no alterations described in IL-17 levels in others. To address this gap in the literature, a systematic review of altered IL-17 levels in rodent models of ASD was conducted. In total, 28 studies that explored IL-17 levels were included and observed that this cytokine was generally increased among the different models of ASD. The data compiled in this review can help the choice of animal models to study the role of cytokines in the development of ASD, seeking a parallel with immune alterations observed in individuals with this condition.

**Systematic Review Registration:**

PROSPERO, identifier CRD42022306558.

## Introduction

Autism spectrum disorder (ASD) is a complex, heterogeneous neurodevelopmental disorder characterised by restricted patterns of repetitive behaviour, activities, interests and impairments in social skills and communication (1). In Europe, the prevalence was 0.38% in children aged 4 years and 1.55% in children aged 8 years, while in the USA, the prevalence ranged between 1.70% and 1.85%, respectively (2). ASD is also etiologically heterogeneous, and its precise mechanism remains undefined. However, studies have suggested that it results from a combination of immunological, environmental, and genetic factors which can affect brain development and synaptic plasticity (3).

There are currently no biomarkers identified for ASD as its diagnosis is mainly based on clinical assessments (4). However, increasing evidence suggests that an aberrant immune phenotype may be linked to the development of ASD (5). Elevated levels of inflammatory cytokines, such as IL-6, TNF-α, IFN-γ, IL-1β, and IL-12, have been detected in several tissues of individuals with ASD, such as serum, plasma, and brain (6, 7). Elevations in these cytokine levels have been associated with increased stereotypical behaviours, increased aberrant behavioural scores, increased impaired neurodevelopment, and a regressive form of ASD (8, 9). Further studies have also observed increased concentrations of pro-inflammatory cytokines in the amniotic fluid of mothers whose children developed ASD, supporting the role of increased immune activity in ASD development (10, 11).

Another pro-inflammatory cytokine implicated in ASD is IL-17 (12). IL-17 comprises a family of six structurally related cytokines that includes IL-17A (commonly referred to as IL-17), IL-17B, IL-17C, IL-17D, IL-17E, and IL-17F (12). Although IL-17 is well known for its role in pathogenic responses, physiological effects of IL-17 include maintenance of mucosal integrity (13).

Elevated levels of IL-17 have been described in the serum of children with ASD (14), and enrichment in IL-17 genes was reported in individuals with ASD, indicating a possible role for this cytokine in the pathophysiology of this condition (15). Data suggesting the involvement of IL-17 in the development of ASD is also supported by findings in preclinical models of maternal immune activation (MIA) that linked the upregulation of IL-17 during pregnancy to the development of ASD-like behaviours (16).

Animal models have provided ground-breaking data in investigating the relationship between features related to ASD and altered IL-17 levels. It has been demonstrated that inducible models such as MIA by injection of polyinosinic:polycytidylic acid (poly(I:C)) or lipopolysaccharide (LPS), displayed elevated levels of IL-17 in maternal serum followed by upregulation of IL17Ra in foetal brains (16, 17). However, IL-17 levels in foetal, postnatal, and adult offspring of inducible or genetic models of ASD display conflicting data (18–20).

Therefore, a systematic review of data reporting altered IL-17 levels in rodent models of ASD was conducted. We aimed to answer the following questions (1): Are there IL-17 aberrations across rodent models of ASD? (2); If there are IL-17 aberrations across models, what is the magnitude of the difference? (3); Do these IL-17 aberrations differ across the age of rodent models?

## Methods

### Study Selection

Seven electronic databases were utilised for this systematic review: Web of Science, PubMed, BMC, Scopus, SciELO, Proquest Dissertations and Theses — Global, and British Library EthOS. SciELO (South America) was included to address English language bias. The search terms used and the number of records found in each electronic database can be seen in the Supplementary Material (see **Supplementary Table 1**).

We included records containing mouse or rat models of ASD that fitted our inclusion criteria, presenting ASD-like phenotypes and IL-17 analysis. We included all records regardless of the sex or age of the animals. Furthermore, we added all records published between January 1st, 2000, and January 1st, 2022.

We excluded studies that did not analyse IL-17 levels and were not original research studies. Additionally, review articles, posters, and abstracts from conferences, case studies, and retracted articles were excluded.

### Data Extraction, Synthesis, and Quality Assessment

Three reviewers (AJT, LPV, and FDRP) independently screened abstracts, assessed full-text articles, extracted data, and evaluated the risk of bias. Disagreements and inconsistencies were resolved by the consensus of all review group members. The study search strategy is outlined by the Preferred Reporting Items for Systematic Reviews and Meta-Analyses (PRISMA) guidelines. The study is registered on PROSPERO (CRD42022306558).

Data extracted included the type of mouse or rat model, IL-17 alterations, intervention characteristics (i.e. dosing regimen for MIA models), the age, and sex of the animals. Studies were assessed using the CAMARADES’ study quality checklist criteria adapted as follows (21): peer-reviewed publication; random allocation to treatment or control; appropriate animal model (MIA, chemically induced, inbred strain); sex-matched animals; age-matched animals; sample size calculation; compliance with animal welfare regulations; and statement of potential conflict of interests. Each study was given a quality score out of eight points. These criteria were chosen to identify the overall methodological quality of those records.

Furthermore, by amending the quality checklist to include age and sex match animals, this review seeks to ensure that appropriate comparisons between control and treatment groups were made. It is possible that cytokine aberrations in ASD models significantly differ across age and sex and ensuring that animals were age and sex-matched may reduce this bias (**Table 1**).

**Table 1:** Bias assessment and study quality using animal data from experimental studies (CAMARADES) checklist items.

Author	Peer-reviewed publication	Random allocation	Animal model	Sex-matched animals	Age-matched animals	Sample size calculation	Compliance with animal welfare regulations	Statement of conflict of interests	Total
Ozaki et al, 2020 (22)	X		X		X		X	X	5
Arrode-Brusés and Brusés, 2012 (23)	X		X	X	X		X	X	6
Schwartzer et al, 2013 (24)	X		X	X	X		X	X	6
Pendyala et al, 2017 (25)	X		X		X		X	X	5
Xu et al., 2017 (26)	X	X	X		X		X	X	6
Reed et al., 2020 (19)	X	X	X	X			X	X	6
Choi et al., 2016 (16)	X		X				X	X	4
Ahmad et al, 2018 (27)	X		X	X	X		X	X	6
Nadeem et al, 2019 (28)	X		X	X	X		X	X	6
Lammert et al, 2018 (29)	X	X	X	X	X		X	X	7
Yasumatsu et al, 2020 (17)	X		X		X		X		4
Bakheet et al, 2017 (30)	X		X				X	X	4
Ansari et al, 2017 (31)	X		X	X	X		X	X	6
Hsiao et al, 2012 (32)	X		X		X		X	X	5
Zhang et al, 2013 (20)	X		X	X			X	X	5
Luan et al, 2015 (33)	X	X	X	X	X		X		6
Chen et al, 2019 (34)	X		X	X			X	X	5
Gumusoglu et al., 2020 (35)	X			X	X		X	X	5
Afroz et al., 2021 (36)	X	X	X	X	X		X	X	6
Alhosani et al., 2021 (37)	X		X		X		X	X	5
Heo et al., 2011 (38)	X		X	X	X		X	X	5
Jaini et al., 2021 (39)	X		X		X		X	X	5
Kalish et al., 2021 (40)	X		X	X	X		X	X	6
Schwartzer et al., 2017 (41)	X	X			X		X	X	5
Senkal et al., 2021 (42)	X	X		X	X		X	X	6
Shin et al., 2021 (43)	X		X		X		X	X	5
Shimizu et al., 2021 (44)	X		X		X		X	X	5
Kim et al., 2022 (45)	X	X	X	X	X		X	X	7

One reviewer (AJT) assessed the risk of bias and included records using the Systematic Review Centre for Laboratory Animal Experimentation (SYRCLE) (46). This risk of bias tool assessed methodological quality using animal study-specific criteria and was adapted (46) from the Cochrane Risk of Bias tool. For each included study, the parameters for each type of bias were marked as Yes (Y), No (N), or Unclear (U). A final score out of 10 was given to each study (**Table 2**).

**Table 2:** Systematic Review Centre for Laboratory animal Experimentation (SYRCLE) scale for risk of bias.

Author	Sequence generation	Baseline characteristics	Allocation concealment	Random Housing	Blinding(Investigators)	Random (outcome assessment)	Blinding (outcome assessors)	Incomplete outcome data addressed	No selective outcome reporting	No other source of bias	Total Score
Ozaki et al, 2020 (22)	N	Y	U	N	N	U	N	U	Y	Y	3
Arrode-Brusés and Brusés, 2012 (23)	N	Y	N	N	N	N	U	Y	Y	Y	4
Schwartzer et al, 2013 (24)	N	Y	N	N	U	U	U	Y	Y	Y	4
Pendyala et al, 2017 (25)	N	Y	N	N	U	N	U	Y	Y	Y	4
Xu et al., 2017 (26)	Y	Y	N	N	U	Y	Y	Y	Y	Y	7
Reed et al., 2020 (19)	Y	Y	Y	U	Y	U	Y	Y	Y	Y	8
Choi et al., 2016 (16)	N	Y	N	N	U	U	N	U	Y	Y	3
Ahmad et al, 2018 (27)	N	Y	N	N	N	U	N	N	U	Y	2
Lammert et al, 2018 (29)	Y	Y	U	U	Y	U	U	U	Y	Y	5
Yasumatsu et al, 2020 (17)	N	Y	N	N	N	N	N	U	Y	Y	3
Bakheet et al, 2017 (30)	N	Y	N	N	N	U	N	N	U	Y	2
Ansari et al, 2017 (31)	N	Y	N	N	N	N	N	Y	U	Y	3
Hsiao et al, 2012 (32)	N	Y	N	N	N	N	Y	U	Y	Y	4
Zhang et al, 2013 (20)	N	Y	N	N	N	N	N	U	U	Y	2
Luan et al, 2015 (33)	Y	Y	N	U	N	N	N	U	Y	Y	4
Chen et al, 2019 (34)	N	Y	N	N	N	N	N	Y	Y	Y	4
Nadeem et al., 2019 (28)	N	Y	N	N	U	U	Y	U	U	Y	3
Gumusoglu et al., 2020 (35)	N	Y	N	N	Y	U	U	Y	Y	Y	5
Afroz et al., 2021 (36)	N	Y	N	N	N	N	Y	U	Y	Y	4
Alhosani et al., 2021 (37)	N	Y	N	N	N	N	N	U	U	Y	2
Heo et al., 2011 (38)	N	Y	N	N	N	N	N	U	N	Y	3
Jaini et al., 2021 (39)	N	Y	N	N	Y	U	Y	U	Y	Y	5
Kalish et al., 2021 (40)	N	Y	N	N	N	U	N	U	Y	Y	3
Schwartzer et al., 2017 (41)	N	Y	N	N	Y	N	N	N	Y	Y	4
Senkal et al., 2021 (42)	N	Y	N	N	Y	U	Y	U	Y	Y	5
Shin et al., 2021 (43)	N	Y	N	N	N	N	N	U	U	Y	2
Shimizu et al., 2021 (44)	N	Y	N	N	Y	N	Y	U	Y	Y	5
Kim et al., 2022 (45)	N	Y	N	N	Y	N	N	U	Y	Y	4

Y (Yes) = 1 score; N (No) = 0 score; U (Unclear) = 0.

## Results

### Study Selection

**Figure 1:**
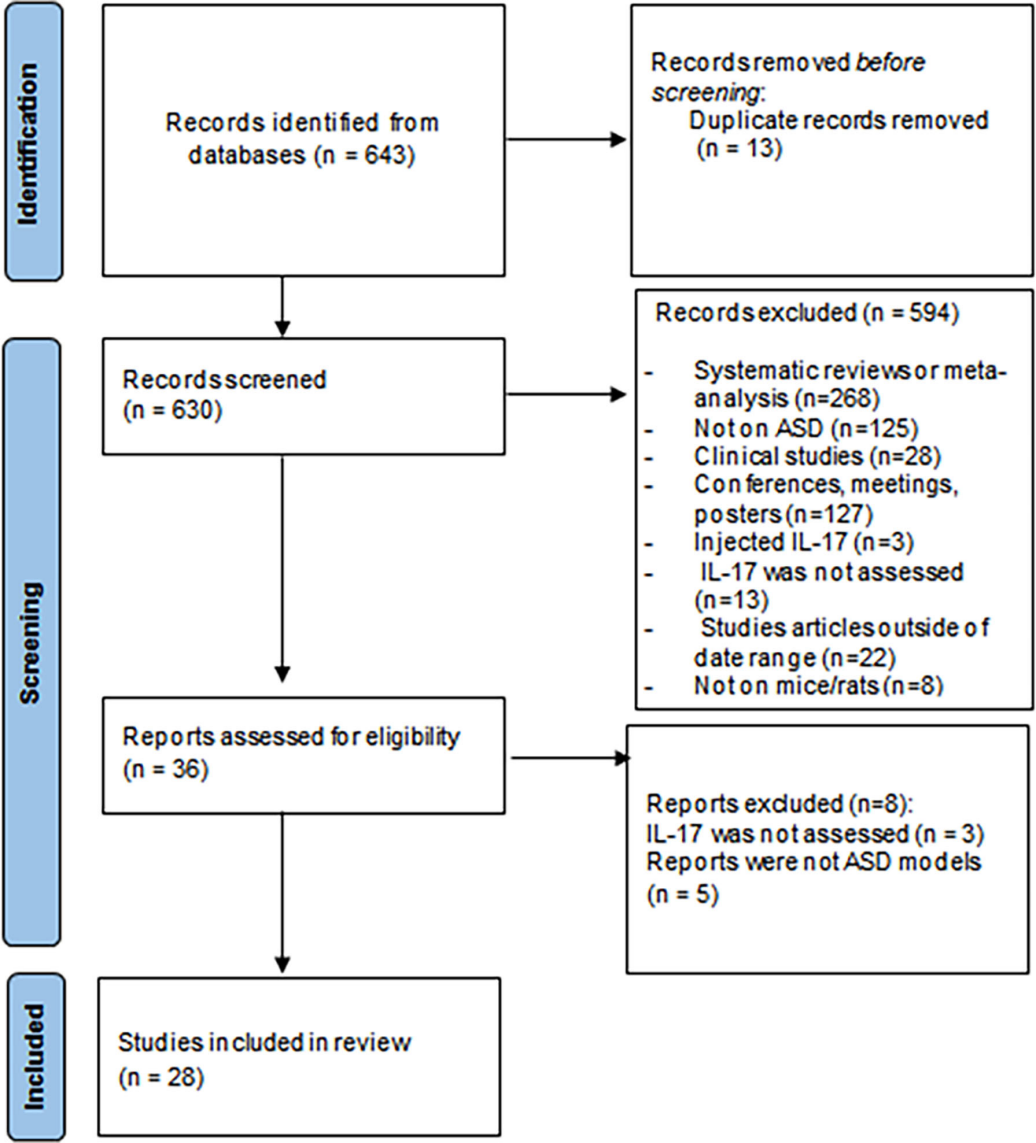
Preferred Reporting Items for Systematic Reviews and Meta-Analyses (PRISMA) flowchart.

A total of 643 records were obtained from the database search (see **Supplementary Table 1**). Following duplicate removal, we screened 630 records, and 28 studies met inclusion criteria (**Figure 1**). All these articles were considered appropriate and were included in this review. Among them, two articles - Chen et al., 2019 and Ozaki et al. (22, 34) - were added using a snowballing technique as they fitted the inclusion criteria but did not show up in the total searches and one article - Kim et al. (45) - was included as it met the inclusion criteria and was published in January, 11 2022. A total of 28 papers were analysed in this review.

Only two studies using rat models matched the criteria for this review (26, 42). Many of the studies generated by the database searches focused on mouse models of ASD. This indicates a data gap and raises the question of if mouse models have better mechanical viability when modelling immune aberrations of ASD compared to rat models; however, this may also be due to the broader availability of mouse cytokine evaluation kits and the prevalence of mouse models in immunology.

### Quality Assessment

According to CAMARADES’ quality scale, the 28 records included articles had a mean score of 5.42 ± 0.77 (mean ± standard deviation) out of a possible 8 (**Table 1**). All papers were published in peer-reviewed journals, and most used models of ASD. Most records used age and sex-matched animals. However, some articles were unclear on whether female rodents were included. This may affect the results obtained since sex-biased alterations of IL-17 in rodent models of ASD is unknown. Furthermore, none of the articles showed the sample size calculations, further affecting the results. Also, the allotment of treatment groups, the use of different litters, and the number of pups per litter were not mentioned. Nevertheless, all articles complied with animal welfare regulations, and many articles included disclosure of potential conflicts of interest.

The assessment of the risk of bias can be seen in **Table 2**. The mean SYRCLE score among all included records was 3.82 ± 1.41. Of the 28 studies included, no papers randomly housed the animals within the animal unit. Most of the records did not blind investigators, and similarly, only 9 papers utilised blinded outcome assessors. In addition, many of the studies did not appropriately generate and apply a sequence generation to the animals used. One study completed a random outcome assessment (26). Similarly, only 8 studies addressed incomplete data.

Furthermore, only 1 of the studies included in this review, Reed et al. (19), concealed the allocation of control and treatment groups (19). All papers had no other apparent sources of bias when reviewed with the SYRCLE guidelines. All studies also established the baseline characteristic of control IL-17 levels compared with ASD rodent models.

### Qualitative Synthesis

#### Altered IL-17 in MIA Models

A total of 16 studies identified from the database search utilised MIA models of ASD, and all of them described alterations in IL-17 levels (16, 17, 19, 22–26, 29, 32, 33, 35, 41, 42, 44) (**Table 3**). Most of the papers used one of two different protocols of inducing MIA, either utilising an intraperitoneal (i.p.) injection of 20 mg/kg of poly(I:C), or an injection of 0.05 mg/kg of LPS, at embryonic day (E) 12.5. One study used a subcutaneous osmotic pump releasing IL-17 at a rate of 0.025 mg/kg per hour to simulate chronic inflammation (35). A further study induced the MIA model through sensitisation with 10µg of ovalbumin to mimic chronic maternal allergic asthma in C57BL/6 and FVB mice, which develop mild and a more severe asthma respectively (41). Out of the 15 studies, 13 reported increases in IL-17 levels, with one article reporting no alteration in IL-17 production compared to BTBR mice (a well-established model of ASD) (24) and a second report noting a significant decrease in IL-17 at postnatal day 30 (25). A further study found that IL-17 levels decreased at 48 hours post injection (44). IL-17 was assessed at different ages throughout the studies, from foetal to adult (15 weeks old) and in both offspring and dams. When comparing results for each age point, much of the data obtained for each age was similar. There was also a variety of tissues used, the most frequent being offspring serum, brain, kidney, spleen, maternal serum, placenta, and uterus. One study examined IL-17 secretion in lymphocytes from the lamina propria derived from colon tissue (45). Many of the papers that used the same tissue types for the analysis reported similar IL-17 alterations. The studies utilised a variety of techniques, including ELISA, flow cytometry, cytometric bead array, and real-time PCR to assess and infer IL-17 levels.

**Table 3:** Qualitative synthesis of studies included.

Author	Species	Strain	Sex	Sample size	Rodent ASD model	Dose (mg/kg)	Inducement	Route	Treatment age	Testing age	Tissue used	Technique used	IL-17 alteration	Statistically significant
Ozaki, 2020 (22)	mice	C57BL/6N	male	4	MIA	10	Poly(I:C)	i.p	E12, E15	foetal, E12,E15, P10	microglia, liver, placenta, brain, serum	RT-PCR,	increase	No
Arrode-Brusés, 2012 (23)	mice	C57BL/6N	both	NS	MIA	20	Poly(I:C)	i.p	GD16, PND4	P4, foetal	maternal serum, offspring brains	Milliplex Map	increase	Yes
Schwartzer, 2013 (24)	mice	C57BL/6N /BTBR	both	12 to 18	MIA/BTBR	20	Poly(I:C)	i.p	E12.5	11 weeks	spleen	murine multiplexing bead immunoassays	increase	Yes
Pendyala, 2017 (25)	mice	(Pcp2-EGFP)BT153Gsat/Mmmh	both	P1,P7= 4; P14,P30 =6	MIA	20	Poly(I:C)	i.p	E12.5	P1, P7, P14, P30	cerebellar lysates	multiplex ELISA, QAM-CYT-5	decrease at P30	Yes
Xu, 2017 (26)	rats	Wistar	both	10	MIA	0,05	LPS	i.p	E12.5	1 month	serum	ELISA	increase	No
Reed, 2020 (19)	mice	C57BL/6N	male	4 to 8	MIA	20	Poly(I:C)	i.p	E12.5	E12.5	serum	ELISA	Increase after immune stimuli	Yes
Choi, 2016 (16)	mice	C57BL/6N	male	NS	MIA	20	Poly(I:C)	i.p	E12.5	E14.5	placenta, decidua, maternal serum	ELISA, RT-PCR, Flow cytometry	increase	Yes
Ahmad, 2018 (27)	mice	n/a	male	NS	BTBR	n/a	n/a	n/a	n/a	7-9 weeks	spleen, brain	Flow cytometry, western blotting, RT-PCR	increase	Yes
Nadeem, 2019 (28)	mice	n/a	male	NS	BTBR	n/a	n/a	n/a	n/a	8-10 weeks	Splenocytes, CD4+ cells, cerebellum	Flow cytometry, RT-PCR	increase	Yes
Lammert, 2018 (29)	mice	C57BL/6N	both	NS	MIA	20	Poly(I:C)	i.p.	E11.5, E12.5	E14.5	maternal serum	ELISA, Cytokine blockade	increase	Yes
Yasumatsu, 2020 (17)	mice	C57BL/6N	male	NS	MIA	0.05	LPS	i.p	E14	foetal, mother	maternal uterus, brain	ELISA, qRT-PCR, Flow cytometry	increase	Yes
Bakheet, 2017 (30)	mice	n/a	male	NS	BTBR	n/a	n/a	n/a	n/a	6-8 week	spleen, brain, CD4 t cells	Flow cytometry, RT-PCR, intracellular staining	increase	Yes
Ansari, 2017 (31)	mice	n/a	male	6	BTBR	n/a	n/a	n/a	n/a	6-8 week	spleen, brain	Flow cytometry, western blotting, RT-PCR	increase	Yes
Hsiao, 2012 (32)	mice	C57BL/6N	both	NS	MIA	20	Poly(I:C)	ip	E12.5	15 weeks	CD4+ T cells	ELISA	increase	Yes
Zhang, 2013 (20)	mice	BTBR/C57BL/6	both	NS	BTBR	n/a	n/a	n/a	n/a	2-3 months	Splenic TCD4	Flow cytometry	increase	Unclear
Luan, 2015 (33)	mice	B6	both	NS	MIA	0,05	LPS	i.p	E12.5	dam, 8-12-week offspring	CD4 T cells from liver and, spleen	Intracellular cytokine staining	increase	Yes
Chen, 2019 (34)	mice	C57BL/6N	male	5	Chemical and MIA	0.25 μl/hr 20	BDE209, Pb, BDE209/Pb poly(I:C)	subcutaneous osmotic pump i.p	E9.5 E12.5	P 30	serum	Cytometric bead array	increase	Yes
Gumusoglu, 2020 (35)	mice	C57BL/6N	both	8	MIA	25	IL-17	subcutaneous osmotic pump	chronic (hourly)	E18	kidney, placenta, brains	ELISA	increase	No
Afroz, 2021 (36)	mice	C57Bl6	Female	8 -9 per group	Parental High salt diet (HSD)	chow supplemented with 0.1% NaCl	8 weeks HSD	Oral	3-4 weeks		Maternal serum	Flow Cytometry	No	No
Alhosaini, 2021 (37)	mice	C57 /BTBR	Male	6 per group	BTBR	n/a	n/a	n/a	n/a	10-12 week	Splenocytes, Brain Tissue	Flow Cytometry, RT-qPCR, Western Blotting	Increase in BTBR	Yes
Heo, 2011 (38)	mice	C57BL/6 / BTBR/ BCF1/CBF1	both	3 - 5 per group	BTBR/ BCF1/CBF1	N/A	N/A	N/A	N/A	PND 21 and 70	Liver	ND	Increase	Yes ** data not shown
Jaini, 2021 (39)	mice	PtenWT/m3m4 and PtenWT/WT	Female	5 per group	PtenWT/m3m4 and PtenWT/WT	N/A	N/A	N/A	N/A	Pregnant dams at E17.5	Maternal Spleen/Serum	qRT-PCR, ELISA	No	n/a
Kim, 2022 (45)	mice	C57Bl6	both	12 - 18 (Variable)	MIA	20 mg/kg	poly(I:C)	i.p.	E12.5	8-10 weeks	Offspring Serum, colonic lymphocytes	Flow cytometry	Increase after immune challenge	yes
Sekal, 2021 (42)	Rat	Sprague Dawley	both	10 per group	Neurodevelopment disorder induced by hyperosmotic consumption by mothers	5 mL/kg	21 days	Oral Gavage	n/a	PND 50	offspring brain homogenates	Elisa	Increase	yes
Schwartzer 2017 (41)	mice	C57 and FVB/Ant	Female	12-17	OVA-induced MIA	1% (wt/vl)	aerosolised solution of 1% (wt/vl) OVA in PBS	aerosolised	Gestational days 9.5, 12.5, and 17.5	On gestational day 17.5	Maternal serum	Flow cytometry	Increase in C57 pregnant dams exposed to OVA	yes
Shimizu, 2021 (44)	mice	C57BL/6J	both	8-9 per group	MIA	20 mg/kg during pregnancy and 20 mg/kg or 4 mg/kg postnatally	poly(I:C)	i.p.	E12.5, 15.5 and 16.5. Pups also received poly(I:C) injections at 3-4 weeks old	3-4 weeks	Offspring serum	ELISA	Increase in MIA-offspring and MIA-offspring that received postnatal injections of poly(I:C)	yes
Shin, 2021 (43)	mice	BALB/c hairless mice	both	9 - 13	VPA	600 mg/kg	VPA	i.p.	E12.5	PND 1, 4, 12 and 21	Offspring blood, skin and brain	ELISA	Increase on the skin on PND1, 4 and 21 and brain in PND1	yes
Kalish, 2021 (40)	mice	C57BL/6	both	8	MIA	20 mg/kg	Poly(I:C)	i.p.	E12.5	E14.5 and 18.5	Foetal cortex	No measurement of IL-17	IL-17 blockage led to the suppression of integrated stress responses in males	yes** alterations are dependent on IL-17

***Observation.

#### Altered IL-17 in Chemically Induced Models

One study investigated if exposure to teratogens during pregnancy could induce similar alterations to the well-established model of maternal activation poly(I:C) (34). In this case, pregnant C57BL/6 mice were exposed to the polybrominated diphenyl ether (PBDE) 209 and lead (Pb), common pollutants that co-exist in the environment. The study suggested that the perinatal exposure to the mixture of PBDE209 and Pb elicited restricted, repetitive patterns of behaviour and affected learning in male offspring, and IL-17 levels were increased in the serum of mice at postnatal day 30 (34). However, social behaviour was not impaired after chemical exposure, differently from the observed in the poly(I:C) model (34). This suggests that although not all behavioural alterations typically associated with animal models of ASD were induced by these teratogens, neurobehavioral alterations were still present and therefore presenting relevant validities as a model. A further study found that neonatal and P1 pups from dams treated with valproic acid to induce chronic immune response had significantly higher IL-17A in both the skin and brain. These IL-17A increases were also correlated to ASD-like features, as examined through the Morris Water Maze (43).

#### Altered IL-17 in Inbred Models

A total of 8 studies utilised inbred rodent models of ASD, and 6 of them found alterations in IL-17 concentrations (20, 24, 27, 28, 30, 31). All papers used the BTBR mice, with five of the eight papers only using males. However, some studies used a smaller age range of mice than the papers that used the MIA models, with a range of 6-13 weeks. Out of the eight studies that observed IL-17 aberrations, all found a significant increase in IL-17 intracellular levels and mRNA expression (**Table 3**). One paper found a significant increase in IL-17A intracellular staining in T CD4+ cells from the spleen, as well as increase in IL-17A mRNA expression and protein expression in brain tissue when compared to C57BL/6 mice (37).

A 2011 study by Heo et al. found that 3 days following infection with Keyhole limpet haemocyanin (KLH) and *Listeria monocytogenes*, BTBR mice had significantly increased levels of IL-17 in the liver when compared to C57BL/6 mice. However, the data regarding IL-17 alterations found in this study is not shown, making it difficult to fully understand and evaluate the immune alterations related to IL-17 (38).

Interestingly, BTBR mice, which present higher basal levels of IL-17 when compared to controls, were described to increase these levels even further when exposed to perinatal poly(I:C), suggesting that BTBR mice may have a dysregulated cytokine response that could impact the ASD-like behavioural alterations (24).

All 8 studies utilised similar tissues including the spleen and brain, mostly focusing on investigating intracellular levels of IL-17 in splenocytes from BTBR mice, as well as IL-17 expression in brain tissue. Techniques varied from ELISA assays, flow cytometry, Western blotting, and real-time PCR to assess IL-17 concentrations.

#### Altered IL-17 in Genetic Models

A total of 2 studies utilised genetically modified rodent model of ASD (19, 39). Reed et al. (19) utilised mice models in which the animals harbour mutations in genes related to an increased chance of ASD, including contactin-associated protein-like 2 (Cntnap2), fragile X mental retardation-1 (Fmr1), and multiple ankyrin repeat domains 3 (Shank3) genes, for comparison with environmental ASD model such as MIA. This paper showed that inflammatory response caused by LPS injection on offspring born to mothers injected with poly(I:C) at embryonic day (E)12.5 (MIA-offspring) rescued social behaviour deficits, and that this LPS-induced rescue is dependent on IL-17A. However, this same result was not observed in the monogenic models of ASD, suggesting that differences in cytokine production across models are instrumental to the behavioural rescue observed. This hypothesis was further supported since after LPS injection both (a) the monogenic mice did not display an increase in plasmatic IL-17A and (b) IL-17A blockage in the MIA-offspring mice prevented the sociability rescue (19).

Jaini et al. (39) utilised *Pten^WT/m3m4^* mouse dams to breed 2 offspring genotypes, *Pten^WT/m3m4^* and *Pten^WT/WT^*, which were then bred to produce offspring. PTEN mutations are associated with ASD, accounting for 2% of all ASD and 17% of macrocephalic-ASD cases (39). Inflammatory markers, cellular phenotypes and gene expression were examined in the dams and offspring *via* multiplex ELISA, multiplex bead-based cytokine assays and gene expression panel analysis on E17.5. This paper found no IL-17A transcripts in the spleen from either mouse genotype used in this study and no differences in IL-17A in the serum were found (39).

#### Altered IL-17 in High Salt Diet Model

A total of 2 papers used a model in which animals were fed diets that included parental high salt consumption in an attempt to induce the development of ASD-like characteristics in the offspring. Afroz et al. (2021) used 8% NaCl in chow and an additional 1% NaCl in drinking water for male and female C57BL/6 mice for 8 weeks, after this period of time animals were used for breeding (36). IL-17A was detected on maternal serum without any alterations as compared to controls using flow cytometry-based assay (36). Another study exposed pregnant Sprague-Dawley rats to three different hypertonic solutions, including 3% NaCl, mineral water (3% NaHCO3+magnesium+calcium content) and Ayran (0.8% NaCl content) (42). Pregnant rats were exposed to hypertonic solutions throughout the pregnancy (21 days) *via* oral gavage at a dose of 5 mL/kg with dosing repeated up to 3 times within 24 h period (42). IL-17A increase was detected in the brain homogenate from male offspring of hypertonic solution fed-rats *via* ELISA kit (42).

## Discussion

To our knowledge, this is the first systematic review assessing IL-17 levels in rodent models of ASD. Our findings show that IL-17 levels are increased in different tissues and in the serum of ASD rodent models, as reported by 23 out of 28 studies included in this review. Also, increased IL-17 levels could be observed in pregnant mice, embryos, and offspring, highlighting the possible role of this cytokine during neurodevelopment, pathogenesis and pathophysiology of ASD, as well as in behavioural alterations.

This review has also highlighted the potential role of IL-17 in the establishment of ASD-like behaviours across rodent models with a specific focus on offspring and maternal IL-17 levels. The studies examined in this review have demonstrated the role of the maternal immune state and the increase in IL-17 production in the development of ASD-like features. Several papers stressed the importance of the maternal immune state and how it relates to microbiota (29, 45), genetics (39), and nutrition (36).

Several papers have suggested the important role of homeostatic maternal immune state is and the development of ASD (11, 16, 22). Whereas other studies found increased pro-inflammatory cytokine levels in the amniotic fluid of mothers whose children develop ASD, including IL-6, a cytokine necessary for the differentiation of Th17 cells (10, 47).

As previously discussed in this review, much of the increased IL-17 production appeared to be derived from Th17 cells (27, 28, 30, 31). However, another possible producer of IL-17 in ASD models seems to be γδ T cells. Increased concentrations of IL-17 producing γδ T cells were observed in the uterus of pregnant mice after MIA (17). Previous data have shown that residential IL-17 producing γδ T cells in the meninges of the brain can control anxiety-like behaviour in mice *via* IL-17 receptor A signalling (18, 48). Other studies found that increased meningeal γδ T cells reduce synaptic density *via* IL-17 pathways (48). Taken together, these findings suggest that whilst the increase of uterine γδ T cells may induce a systemic increase of IL-17 in pregnant mice, the increase may cause a localised inflammatory response in the brain of their offspring, which may affect synaptic density (48). However, uterine γδ T cells may not be the only source of upregulated IL-17. Kim et al. (49) have demonstrated that T cells isolated from gut connective tissue of poly(I:C) treated mice expressed increased levels of IL-17A when compared to tissue from PBS treated mice (49). This study offers insightful data relating to the role of the gut in ASD mouse models. This study, however, did not match the inclusion criteria and was not included in the present systematic review.

In the gut, another factor potentially playing a role in the development of ASD-like features is maternal microbiota. Lammert et al. (29) found that C57BL/6 mice from Taconic Biosciences (Tac) displayed higher levels of IL-17A after poly(I:C) injections when compared to C57BL/6 Jackson Laboratory’s (JAX) mice. They also presented a high relative abundance of segmented filamentous bacteria (SFB), known to promote IL-17A inflammatory response (29). Offspring from poly(I:C) treated Tac mice displayed ASD-like behavioural alterations. However, those alterations were not observed in saline controls or in the offspring of JAX mice. Additionally, experiments of co-housing were able to induce behavioural alterations in JAX mice offspring and led to an increase in IL-17A in maternal serum after poly(I:C) injection, further relating the role of the gut microbiota in the production and regulation of IL-17 and Th17 cells (49). Those findings are also supported by Choi et al. (16), who demonstrated that upregulation of IL-17A after poly(I:C) led to abnormal cortical development in the offspring as well as ASD-like altered behaviour (16). Those alterations were reversed after pre-treatment with IL-17A blocking antibodies, reinforcing the hypothesis that altered maternal IL-17A response plays an important role in the development of ASD in the offspring (16). Furthermore, data from Kim et al. (2022) (45) have demonstrated that MIA immune alterations observed in the offspring may happen postnatally, as crossfostering pups from PBS-treated mothers with MIA mothers led to an increase in the percentage of colonic IL-17A and IFN- γ -producing Th17 cells in the pups after infection (37). This immune priming seems to be related to the maternal microbiota as experiments with stool transfer have demonstrated that offspring from animals that received stool transfer from MIA-mothers displayed pro-inflammatory immune phenotype after immune challenge (45).

Clinical evidence also supports that microbiota plays a role in the pathogenesis and development of ASD. It has been demonstrated that children with ASD displayed a decrease in anti-inflammatory *Faecalibacterium* bacteria and an increase in the pathogenic inflammatory *Enterobacteriaceae* and *Sutterellaceae* bacteria in the faecal microbiome (50). A further study found that an oral administration of the endogenous human micro-organism, *B. fragilis*, improved the behavioural ASD-like features in mouse models as well as metabolite modulation in the gut (51).

In line with alterations induced by maternal microbiota, data resulting from altered diet and the development of ASD-like features in the offspring has also been debated (36, 42). Previous reports have demonstrated that excess dietary salt leads to cognitive impairment, increased Th17 differentiation in the intestine and IL-17 plasmatic levels in mice (52). This impairment is dependent on lymphocytes, as it was not observed in mice lacking IL-17 or Rag1^−/−^ mice (52). However, Afroz et al. (2021) have demonstrated that a high salt diet (HSD) did not induce any behavioural or immune alteration in HSD-fed mice. Furthermore, behaviour alterations were present in the offspring of HSD-fed mice, including altered social behaviour and increased repetitive behaviours (36, 52). In another paper analysed in this review, Senkal et al. (2021) (42) have demonstrated that male offspring rats from animals exposed to hypertonic solutions during pregnancy displayed a pro-inflammatory immune profile in brain tissue with increased IL-17 as well as ASD-like behaviours, reinforcing the hypothesis that maternal state in regards to diet and microbiota may play a role in the development of ASD-like characteristics in the offspring (42).

The kinetics of induction of MIA may play a pivotal role in the development and severity of ASD-like features in rodent models, with alteration of IL-17 levels being associated with specific points of synaptogenesis (25). Synaptic genes were also enriched in the brains of the offspring (35), suggesting a link between the MIA induction time point, maternal and foetal IL-17 levels, and synaptic development, which may induce autistic-like behaviour and affect the immune phenotype (53, 54). However, further studies are necessary to confirm the association between the aberrant IL-17 levels and unregulated synapses seen in ASD.

ASD has a strong genetic component, as demonstrated by high heritability amongst siblings (55). Another evidence for the genetic impact of ASD comes from single gene alterations linked with developmental disorders such as mutations in FMR1 (fragile X syndrome), MECP2 (Rett syndrome), TSC1/TSC2 (tuberous sclerosis complex), and CACNA1C (Timothy syndrome), as well as mutations in CNTNAP2 (cortical dysplasia–focal epilepsy syndrome) (55, 56). Animal models with single gene alterations allow for the understatement of how those mutations play a role in the development of the core characteristics of ASD, such as behavioural alterations (56). However, as it was described by Reed et al. (19), animal models for Cntnap2, Fmr1, and Shank3 did not display any IL-17 alteration after immune challenge with LPS as compared to the MIA-offspring model, indicating that immune alterations may be related to environmental factors or alteration in several genes (19). In this regard, Jaini et al. 2021 (39) hypothesised that maternal genetics may be an important modulator of neuro-immune alterations in the offspring and using the mice model for mutation in PTEN (Pten^WT^/^m3m4^), which has also been associated with ASD, set to investigate the influence of maternal genetics on ASD development in offspring (39). However, no IL-17A transcription was detected on the spleen of pregnant mice, nor any difference in the cytokine was detected in serum (39). Nevertheless, the authors demonstrated that low IL-10 during pregnancy was directly correlated with decreased complement expression in the foetal liver and offspring from mutant mice also displayed altered social and repetitive behaviours without external immune insult (39).

Several limitations should be highlighted when describing our analyses. We were not able to perform a meta-analysis due to the high methodological heterogeneity among studies, including diverse outcomes, distinct species, experimental designs, and types of animal models. Most of the studies analysed in this systematic review used male mice or did not explicitly examine the differences between the sexes. Although this is understandable due to the increased prevalence of ASD in male individuals, excluding one sex can hinder the translational validity of the results (57, 58). The exclusion of female-specific results could potentially lead to a data gap regarding immune aberrations in females, which could affect research and potential future treatments.

Another aspect that could affect IL-17 levels is receptor desensitisation due to persistent stimulation (59). Further studies are necessary to examine how IL-17 receptors could be altered in rodent models of ASD and how their altered signalling can affect pathways dependent on IL-17, such as mRNA stabilisation *via* the ACT1 dependent pathway (60).

IL-17 production and the effects of IL-17 have been noted to change with age within the papers examined in this review. The study by Pendyala et al. showed that although IL-17 was altered at all stages of development, the aberration was only significant at postnatal day 30 when IL-17 was decreased (25). Equivalent results were found with IL-17 concentrations significantly up-regulated by MIA after poly(I:C) exposure at gestational day 17 followed by lower levels at postnatal day 5 in the study by Arrode-Brusés and Brusés (2012) (23). These results suggest that MIA primes offspring’s immune system for a pro-inflammatory response following secondary exposure to poly(I:C), and IL-17 levels may be altered with age. However, this requires further exploration in both clinical and preclinical settings.

Additionally, the technique used to measure cytokine levels, which can changed between the studies, may play a role in the observed outcome. While some methods used in the papers, i.e. ELISA and flow cytometry, measure IL-17 protein levels, qRT-PCR measures IL-17 mRNA levels. Many of the studies that utilised qRT-PCR also included a technique to measure the IL-17 protein concentration, such as in a study where ELISA assays and flow cytometry were used to measure protein IL-17 and IL-17 producing cells alongside RT-PCR (16). One study confirmed the presence of elevated levels of IL-17 in BTBR mice by intracellular staining. However, they did not provide the data demonstrating such increase, mainly pointing out that even though there were elevated levels of IL-17, there was no differentiation into a Th17 profile (20). It is difficult to understand the full scope of IL-17 alterations in the model used in this work, as some data was not shown in the article; this absence of data also makes it difficult to incorporate the findings of this article in a meta-analyses. In despite of that, the study was included in this systematic review as it met all inclusion criteria.

In summary, this systematic review identified common alterations in IL-17 across different models of ASD and almost the totality of the paper reported increases in the level of IL-17 or in IL-17 producing cells, across different detection techniques. The selection of the papers and parameters analysed may help the choice of animal models to study the role of IL-17 and other cytokines in the development of ASD and/or behavioural alterations. It is important to notice that, although the results were consistent, there is a need for further characterisation of the meaning of IL-17 increase, and its importance to the pathophysiology of ASD.

## Data Availability Statement

The original contributions presented in the study are included in the article/**Supplementary Material**. Further inquiries can be directed to the corresponding authors.

## Author Contributions

Conception and design of the work: AT, DM-d-C, VB-J, IR. Acquisition, analysis, and interpretation of data for the work: AT, LV, and FR-d-P. Drafting of the work: AT, LV, and FR-d-P. Revision: FR-d-P, DM-d-C, IR, VB-J. Final approval of the version to be published: DM-d-C, IR, VB-J. All authors contributed to the article and approved the submitted version.

## Funding

This study was supported by the University of Central Lancashire, UK; the Oswaldo Cruz Foundation, the National Institute of Science and Technology on Neuroimmunomodulation (INCT-NIM, CNPq), the Rio de Janeiro Research Network on Neuroinflammation (RENEURIN - Faperj), Brazil; the MercoSur Fund for Structural Convergence (FOCEM); and the ERASMUS+ programme.

## Conflict of Interest

The authors declare that the research was conducted in the absence of any commercial or financial relationships that could be construed as a potential conflict of interest.

## Publisher’s Note

All claims expressed in this article are solely those of the authors and do not necessarily represent those of their affiliated organizations, or those of the publisher, the editors and the reviewers. Any product that may be evaluated in this article, or claim that may be made by its manufacturer, is not guaranteed or endorsed by the publisher.
