# Supplementary material for: Aberrant IL-17 Levels in Rodent Models of Autism Spectrum Disorder: A Systematic Review
Source: Front Immunol. 2022 Jun 10;13:874064. doi: 10.3389/fimmu.2022.874064 (PMC9226456; doi:10.3389/fimmu.2022.874064)
Supplement: Supplementary file 1 [file Table_1.docx]

**Supplementary Table 1.** Search terms used and the number of records in each electronic databases

| **Database** | **Search Strategy** | **Number of records** |
| --- | --- | --- |
| Pubmed | ASD AND mouse models AND IL-17  Austism Spectrum Disorder AND mouse models AND IL-17  ("Autism spectrum disorder" OR "ASD" OR "autism") AND ("mouse model" OR "rat model") AND ("IL-17") | 16  19  8 |
| Proquest | ("Autism spectrum disorder" OR "ASD" OR "autism") AND ("mouse model" OR "rat model") AND ("IL-17") AND Article NOT (Literature Review AND Review AND Conference AND News AND Case Study AND Report AND 2000-01-01 - 2022-01-01 | 531 |
| BMC | ("Autism spectrum disorder" OR "ASD" OR "autism") AND ("mouse model" OR "rat model") AND ("IL-17") | 49 |
| SciElo | ("Autism spectrum disorder" OR "ASD" OR "autism") AND ("mouse model" OR "rat model") AND ("IL-17") | 0 |
| British Library EThoS | (("Autism spectrum disorder" OR "ASD" OR "autism") AND ("mouse model" OR "rat model") AND ("IL-17") | 0 |
| Scopus | ("Autism spectrum disorder" OR "ASD" OR "autism") AND ("mouse model" OR "rat model") AND ("IL-17") | 9 |
| Web of Science | ("Autism spectrum disorder" OR "ASD" OR "autism") AND ("mouse model" OR "rat model") AND ("IL-17") | 11 |
